# Sulforaphane Targets TRA-1/GLI Upstream of DAF-16/FOXO to Promote *C. elegans* Longevity and Healthspan

**DOI:** 10.3389/fcell.2021.784999

**Published:** 2021-12-03

**Authors:** Huihui Ji, Zhimin Qi, Daniel Schrapel, Monika Le, Yiqiao Luo, Bin Yan, Jury Gladkich, Michael Schaefer, Li Liu, Ingrid Herr

**Affiliations:** Molecular OncoSurgery, Section Surgical Research, Department of General, Visceral and Transplant Surgery, University of Heidelberg, Heidelberg, Germany

**Keywords:** sulforaphane, aging, *C. elegans*, TRA-1/GLI, DAF-16/FOXO

## Abstract

Broccoli-derived isothiocyanate sulforaphane inhibits inflammation and cancer. Sulforaphane may support healthy aging, but the underlying detailed mechanisms are unclear. We used the *C. elegans* nematode model to address this question. Wild-type and 4 mutant *C. elegans* worm strains were fed in the presence or absence of sulforaphane and *E. coli* food bacteria transfected with RNA interference gene constructs. Kaplan–Meier survival analysis, live imaging of mobility and pharyngeal pumping, fluorescence microscopy, RT–qPCR, and Western blotting were performed. In the wild type, sulforaphane prolonged lifespan and increased mobility and food intake because of sulforaphane-induced upregulation of the sex-determination transcription factor TRA-1, which is the ortholog of the human GLI mediator of sonic hedgehog signaling. In turn, the *tra-1* target gene *daf-16*, which is the ortholog of human FOXO and the major mediator of insulin/IGF-1 and aging signaling, was induced. By contrast, sulforaphane did not prolong lifespan and healthspan when *tra-1* or *daf-16* was inhibited by RNA interference or when worms with a loss-of-function mutation of the *tra-1* or *daf-16* genes were used. Conversely, the average lifespan of *C. elegans* with hyperactive TRA-1 increased by 8.9%, but this longer survival was abolished by RNAi-mediated inhibition of *daf-16*. Our data suggest the involvement of sulforaphane in regulating healthy aging and prolonging lifespan by inducing the expression and nuclear translocation of TRA-1/GLI and its downstream target DAF-16/FOXO.

## Introduction

Global population aging is a challenge for worldwide health systems. By 2050, the world’s population aged 60 years and older is expected to reach two billion (World Health Organization and Ageing and health, 2018). Life expectancy is increasing because of improved medical care and hygienic standards, infection control interventions, antibiotics, vaccinations, and others. However, the elderly are not necessarily healthier, and many do not match the WHO aims to maintain functional ability that enables wellbeing in older age (Crimmins, 2015; Olshansky, 2018; World Health Organization and Ageing and health, 2018). Aging today is reflected by lifestyle diseases such as heart diseases, diabetes, and cancer (Niccoli and Partridge, 2012). A high correlation between cancer and aging occurs because the incidence of cancer increases sharply with age (Sedrak et al., 2021), such as pancreatic ductal adenocarcinoma, one of the deadliest malignant tumors (Sohal et al., 2016; Neoptolemos et al., 2018; Siegel et al., 2021). Therefore, several lifestyle and nutritional strategies aim to promote healthy aging to lower biological age and thereby cancer risk.

Epidemiological studies have indicated that the regular consumption of broccoli and other Brassicaceae family vegetables, such as Brussel sprouts, cabbage, cauliflower, kale, swede, and turnip, can reduce the incidence of many cancer types, including pancreatic cancer (Chan et al., 2005; Heinen et al., 2012). In particular, the bioactive, anti-inflammatory isothiocyanate sulforaphane, which is abundant in broccoli, is particularly well studied (Herr and Büchler, 2010). Sulforaphane acts antioxidative and inhibits insulin/IGF-1 signaling in the nematode *Caenorhabditis* (*C.) elegans* (Santín-Márquez et al., 2019; Qi et al., 2021). Additionally, sulforaphane reduces hepatic glucose production, improving glucose control in patients with type 2 diabetes (Axelsson et al., 2017). Recent data even showed that a broccoli diet increased the lifespan of the red flour beetle *Tribolium castaneum* by 30% (Grünwald et al., 2013). More specifically, crude extracts of *Brassica chinensis*, also known as pak choy, enhanced the antioxidant activity in a cell-free system and exerted antiaging effects in *C. elegans* (Chen et al., 2016). These data suggest a role of sulforaphane in the metabolic regulation of aging, according to the criteria underlying metabolic aging reviewed by Finkel et al., 2015 (Finkel, 2015).

The nonpathogenic nematode *C. elegans*, originally isolated from garden soil, is a widely used model organism to study aging mechanisms (Shen et al., 2018). The *C. elegans* genome is completely sequenced (Consortium, 1998), and 60–80% of human gene homologs in *C. elegans* have been identified (Kaletta and Hengartner, 2006). *Also,* maintaining the nematodes in the laboratory is inexpensive and straightforward, no ethical regulations have to be followed, the worm body is transparent, individual cells can be observed well under the microscope, and large population/lifespan studies on aging processes can be completed within approximately 30 days (Zhang et al., 2020). Many *C. elegans* genes are related to human disorders, and several stable, transgenic altered *C. elegans* strains with gene mutations are available for a small contribution toward expenses from the *Caenorhabditis* Genetics Center (CGC) at the University of Minnesota, United States (Ashrafi et al., 2003; Watson et al., 2013). Likewise, *C. elegans* genes can be inhibited effectively, rapidly, and specifically by RNA interference (RNAi) because this approach only requires feeding RNAi-transfected *E. coli* food bacteria to the worms.

Most *C. elegans* worms are self-fertilizing hermaphrodites (XX) that are actually females capable of sperm production (Corsi et al., 2015). Adult male *C. elegans* worms (XO) are smaller and thinner than hermaphrodites, behave differently, and have a ventral gonad associated with spicules at the tail for mating. Hermaphrodites have a significantly longer lifespan than males that is controlled by the sex-determination pathway and its terminal transcription factor TRA-1 (Hodgkin, 1987; Pires-daSilva and Sommer, 2004; Grote and Conradt, 2006; Ellis and Schedl, 2007). TRA-1 protein levels are much higher in hermaphrodites than in males, and TRA-1 activity is sufficient to promote the development of hermaphrodites, whereas low TRA-1 activity leads to male development (Schvarzstein and Spence, 2006). Three *fem* genes directly regulate the expression of *tra-1*. Loss of function of *fem-3* results in the hyperactivity of TRA-1 expression (Szabó et al., 2009; Kutnyánszky et al., 2020) and promotes the development of hermaphrodites (Starostina et al., 2007). TRA-1 is orthologous to the human GLI protein, a mediator of Sonic hedgehog signaling, which is involved in embryonic limb development (Briscoe and Thérond, 2013), but also in tumorigenesis (Coquenlorge et al., 2019), and was most recently identified as a suppressor of endometrial stem cell aging (Cho et al., 2019). Interestingly, recent data have indicated that sulforaphane regulates the self-renewal of pancreatic cancer stem cells by modulating the sonic hedgehog–GLI pathway (Li et al., 2013). Findings from another detailed study suggest that TRA-1 mediates aging in a sex-specific way, partly because of the binding of TRA-1 to the *daf-16* locus (Hotzi et al., 2018). DAF-16 is a homolog of the human FOXO transcription factor (Lin et al., 1997) and modulates longevity, stress resistance, development, and resting dauer diapause (Lin et al., 2018; Hou et al., 2019). However, FOXO1 is a tumor suppressor and inhibitor of pancreatic cancer growth (Roy et al., 2010; Pramanik et al., 2014). Thus, the interaction of TRA-1 and DAF-16 in aging and that of their mammalian counterparts in cancer seems likely but requires further clarification.

We investigated whether sulforaphane extends the longevity and healthspan of *C. elegans* via TRA-1-mediated DAF-16 signaling. We performed lifespan assays using wild-type and mutant nematodes or feeding RNAi oligonucleotides to inhibit TRA-1 or DAF-16 expression. We identified a strong relationship between sulforaphane-induced longevity and enhanced expression of TRA-1 and its downstream target DAF-16. These data indicate that sulforaphane promotes female/hermaphrodite development, enhancing the health and longevity of *C. elegans*.

## Materials and Methods

### *C. elegans* Strains

The *C. elegans* wild-type reference strains N2/Bristol (Stiernagle, 2006), CB4270 *tra-1* (e2200) (Zarkower et al., 1994), CB3844 *fem-3* (e2006) *IV* (Hodgkin, 1986), RA7 (rdEx1) (Mathies et al., 2004), and TJ356 *daf-16* (zIs356) *IV* (Lee et al., 2001; Lin et al., 2001) were obtained from the *Caenorhabditis* Genetics Center—CGC (University of Minnesota, Minneapolis, MN, United States), and are summarized in Table 1. All worm strains were maintained at 20°C, except of the CB4270 tra-1 mutant (e2200), which was maintained at 15°C. According to the instructions of the CGC, this *tra-1* mutant needs to be cultured at 15°C to obtain hermaphrodites, because this worm strain will become sterile and gonad-defective at 25°C, due to feminization by smg (NMD) mutations (compare Table 3).

**TABLE 1:** Characterization of *C. elegans* wild-type and mutant strains.

Strain name and WB ID	Genotype	Description	Ref
N2	*wildtype*	Isolated 1951 from mushroom compost in an urban garden by W. L. Nicholas in Bristol. Development time: ∼3 days. Brood size: ∼330 eggs. Lifespan: ∼3 weeks	Stiernagle (2006)
00000001			
CB4270	*tra-1 (e2200)*	Loss-of-function *tra-1* mutant. *Tra-1* is the main effector of sex-determination in *C. elegans;* it inhibits male development and promotes female development. Hermaphrodite at 15°C, sterile and gonad-defective at 25°C, feminized by smg (NMD) mutations. Made by EMS mutagen	Zarkower et al. (1994)
00004546			
CB3844	*fem-3 (e2006) IV*	*Fem-3* is required for male development and inhibits TRA-1. The *fem-3 (e2006)* null-mutation results in feminization of hermaphrodites at 15°C, which transform into fertile females at 25°C, and maintain at 15°C. Made by the Jonathan A. Hodgkin lab	Hodgkin (1986)
00004512			
RA7	*rdEx1*	GFP::*tra-1* fusion protein. Maintain by picking gravid rollers. Made by the Laura Mathies lab	Mathies et al. (2004)
00031307			
TJ356	*zIs356 IV*	The *daf-16* gene is fused to a GFP reporter and driven by the *daf-16* promoter. Made by the A. Rougvie lab by γ-irradiation. Superficially wild type	Lee et al. (2001); Lin et al. (2001)
00005218			

### Culture and Sampling of *C. elegans*

*C. elegans* was cultured on nematode growth medium (NGM: 0.3% NaCl, 1.7% agar, 0.25% peptone, 5 mg/ml cholesterol, 1 mM KPO_4_, and 1 mM MgSO_4_) in plastic dishes (Greiner Bio-One GmbH, Frickenhausen, Germany), which were seeded with a lawn of *E. coli* OP50 food bacteria (CGC, University of Minnesota, Minneapolis, MN, United States). The *E. coli* OP50 bacteria were cultured overnight at 37°C and diluted to an OD_600_ concentration of ∼1.0 as described previously (Ke et al., 2020). Afterward, 100 µL of the *E. coli* OP50 suspension was plated on 35 mm NGM plates for lifespan assays or 300 µL on 60 mm NGM plates to maintain *C. elegans*. The plates were dried overnight at room temperature. *C. elegans* was maintained at 20°C according to the standard protocol (Brenner, 1974), except for the CB4270 *tra-1* (e2200) and CB3844 *fem-3* (e2006) strains, which were maintained at 15°C.

### Lifespan Assays

Lifespan assays were performed as recently described (Qi et al., 2021). For synchronization, 30 gravid *C. elegans* worms were transferred to new NGM plates supplemented with *E. coli* OP50 food bacteria to lay eggs for 3 h. Next, the worms were picked and washed. After the hatching of early-stage L1 larvae from the eggs, the larvae developed within approximately 40 h to late larval stage L4 and were then transferred to new NGM plates containing OP50 food bacteria and 100 µM sulforaphane (DL-sulforaphane; ≥95%; S4441; Sigma–Aldrich, Manheim, Germany). The control plates contained OP50 bacteria without sulforaphane. Sulforaphane was dissolved in DMSO (AppliChem, Darmstadt, Germany) to a 100 mM stock solution. During the following 9 days, the worms were transferred daily to new NGM plates with OP50 food bacteria ± sulforaphane to avoid new larvae hatching from laid eggs on the old NGM plates to avoid falsifying the results of the lifespan experiments. From Day 10, the transfer of worms to new plates was performed every second day because older worms lay fewer eggs. The transfer was performed under the microscope: a platinum pick (Neolab, Heidelberg, Germany) was sterilized by passing it through the flame of a Bunsen burner (WLD-TEC, Arenshausen, Germany), and the tip was loaded with a glob of OP50 bacteria by gently scraping through the bacterial lawn of the LB agar plate. To select a worm, its body was gently tapped with the pick until the worm adhered to the pick and could be transferred to another plate. Here, the tip of the pick was gently touched to the agar until the worm crawled off the pick. Next, the pick was sterilized for the next round of worm transfer. A worm was considered dead if it stopped moving—even after touching. Worms that exhibited internal hatching or climbed up the wall of the dishes were excluded from the analysis.

### RNA Extraction and Real-Time PCR

Synchronized L4 larvae (*n* = 200/group) were transferred to fresh NGM plates seeded with a lawn of OP50 food bacteria ± sulforaphane (100 µM), and 24, 48, 72, or 120 h later, the worms were collected, washed 3× with M9 buffer (3 g of KH_2_PO_4_, 6 g of Na_2_HPO_4_, 5 g of NaCl, 1 ml of 1 M MgSO_4_, in 1 L of H_2_O_bidest_) (Stiernagle, 2006), and crushed under liquid nitrogen. According to the manufacturer’s instructions, total RNA was extracted using the RNeasy Mini Kit (QIAGEN, Hilden, Germany). Next, the high-capacity RNA-to-cDNA^TM^ Kit (Thermo Fisher Scientific, Hilden, Germany) was applied for reverse transcription. PowerUp^TM^ SYBR^TM^ Green Master Mix (Thermo Fisher Scientific, Hilden, Germany) as used to perform RT–qPCR. A 10 µL reaction mixture contained 2 µL of cDNA, 500 nM of each primer, and 5 µL of PowerUp^TM^ SYBR^TM^ Green Master Mix (2×). PCR was performed using the Applied Biosystem^TM^ 7,500 Real-Time PCR System (Thermo Scientific, Schwerte, Germany) and included denaturation for 2 min at 95°C and 40 cycles of amplification (15 s, 95°C; 15 s, 60°C; 1 min, 72°C). *C. elegans*-specific primers (Table 2) were obtained ready-to-use from Eurofins Genomics (Mannheim, Germany).

**TABLE 2:** Primer sequences used to detect *C. elegans* nematode candidate genes.

*Gene*	Type	Sequence
*tra-1*	F	5′-ATC ACG CAT GGC GTT GTG AG-3′
	R	5′-CGC CTG TAT GAG TTC TCC GG -3′
*daf-16*	F	5′-CCA GAC GGA AGG CTT AAA CT-3′
	R	5′-ATT CGC ATG AAA CGA GAA TG-3′
*act-1*	F	5′-CCA GGA ATT GCT GAT CGT ATG CAG AA-3′
	R	5′-TGG AGA GGG AAG CGA GGA TAG A-3′

F: forward primer; R: reverse primer.

### Western Blot Analysis

Synchronized L4 larvae (*n* = 200/group) of the *C. elegans* reporter strain RA7 were incubated in NGM agar plates with OP50 food bacteria, ±100 µM sulforaphane. After 24, 48, 72, or 120 h, the worms were collected and washed 3× with M9 buffer. The worms were transferred to H_2_O_bidest_ containing a protease inhibitor mix (Sigma–Aldrich, Manheim, Germany) in Laemmli buffer and boiled at 95°C for 5 min, followed by centrifugation for 1 min at 13,000 g. According to the standard protocol (Wueseke et al., 2014), the protein content was not measured, but the proteins were extracted from the same number of live worms per group (n = 200). Next, 50 µL of supernatant with protein was loaded per slot of a 10% SDS polyacrylamide gel, and electrophoresis was performed at 120 V for 2 h. The gels were blotted to Immobilon®-P transfer membranes (Millipore, Billerica, United States) using a wet transfer system (Bio–Rad, Hercules, United States), followed by incubation with rabbit polyclonal anti-GFP antibody (ab290; Abcam, Cambridge, United States), diluted 1:1,000, or mouse monoclonal anti-αTubulin antibody (T5168; Sigma–Aldrich, MO, United States), diluted 1:5,000. Anti-mouse IgG and anti-rabbit IgG secondary antibodies (IRDye® 800CW; LI-COR Biosciences, Lincoln, United States) were diluted at 1:5,000 before use. After washing, the membranes were imaged using an Odyssey® CLx Infrared Imaging System (Li-cor®, Lincoln, United States).

### Fluorescence Microscopy

The *C. elegans* RA7 and TJ356 strains were placed on 2% agarose pads (*n* = 5/pad) and paralyzed by adding 50 µL of a 10 μM levamisole solution (Sigma–Aldrich, MO, United States). Images of the live worms (20/group) were taken using a Leica DMRB fluorescence microscope (Leica, Wetzlar, Germany) equipped with a Thorlabs CS895CU Compact Scientific 8.9 MP color camera (Thorlabs GmbH, Bergkirchen, Germany). The expression levels were quantified by measuring the GFP fluorescence intensity using ImageJ.

### RNAi Application

RNAi-mediated inhibition of *C. elegans* genes was performed as previously described (Kamath et al., 2001). The *tra-1* RNAi and *daf-16* RNAi clones were obtained from the *C. elegans* RNAi feeding library, distributed by Source BioScience (Nottingham, United Kingdom). An RNAi-transfected *E. coli* HT115 (DE3) clone or a nontransfected *E. coli* HT115 (DE3) clone was picked from the delivered agar plates and incubated overnight at 37°C in 5 ml of LB medium containing 50 µg/ml of ampicillin (Sigma–Aldrich, MO, United States). Feeding plates were prepared using standard NGM agar plates supplemented with 1 mM IPTG and 25 µg/ml of carbenicillin (both from Sigma–Aldrich, MO, United States). RNAi-transfected *E. coli* HT115 or nontransfected *E. coli* HT115 control bacteria, 100 µL each, at an OD_600_ concentration of ∼1.0, were seeded onto 35 mm NGM feeding plates and dried overnight at room temperature. Next, synchronized L4 larvae (*n* = 30) were transferred to plates and kept at 20°C for 48 h to allow the intake of RNAi-transfected food bacteria. Afterward, the gravid adult worms were picked and seeded onto new feeding plates with the same bacteria and laid eggs for 3 h. After that, the adult worms were washed. After hatching, *C. elegans* larvae proceeded through four larval stages (L1-L4) until the late larval stage L4/young adult stage and were then transferred to new feeding NGM plates for new experiments.

### Pharyngeal Pumping Assay

The frequency of pharyngeal pumping as a measure of appetite and food intake was determined as previously described (Raizen et al., 2012). Synchronized L4 *C. elegans* larvae (n = 20/group) were maintained on NGM plates containing *E. coli* OP 50 bacteria, RNAi-transfected *E. coli* HT115 bacteria, or nontransfected *E. coli* HT115 control bacteria in the presence or absence of 100 µM sulforaphane. The pharyngeal pumping frequency of the terminal pharyngeal bulb of individual worms was counted 60  sec and 3, 6, 9, and 12 days later under an inverted microscope (Leica, Wetzlar, Germany). Additionally, a differential interference contrast microscope (Ni-E, Nikon, Germany) was used to photograph the pharyngeal structure of *C. elegans*.

### Mobility/Body Bending Assay

Worm mobility was used to reflect the functional state of motor neurons and muscle cells (Mergoud Dit Lamarche et al., 2018), as recently described (Qi et al., 2021). Synchronized L4 *C. elegans* larvae (n = 50/group) were maintained on NGM plates containing *E. coli* OP 50 bacteria or nontransfected or RNAi-transfected *E. coli* HT115 bacteria in the presence or absence of 100 µM sulforaphane. The body bends of single worms were counted for 60 s and 3, 6, 9, and 12 days later, and the number of body bends was calculated as body bends/min. A change in the posterior bulb direction or regular swinging of the head was considered a body bend.

### Statistical Analysis

For Kaplan–Meier survival curves, the *p* values of differences between groups were tested using the log-rank test. Student’s t-test was used to assess differences between two groups. IBM SPSS Statistics 25 software was used to perform statistical analysis. *p* < 0.05 was considered statistically significant.

## Results

### TRA-1 Is Required for Sulforaphane-Induced Longevity

**FIGURE 1:**
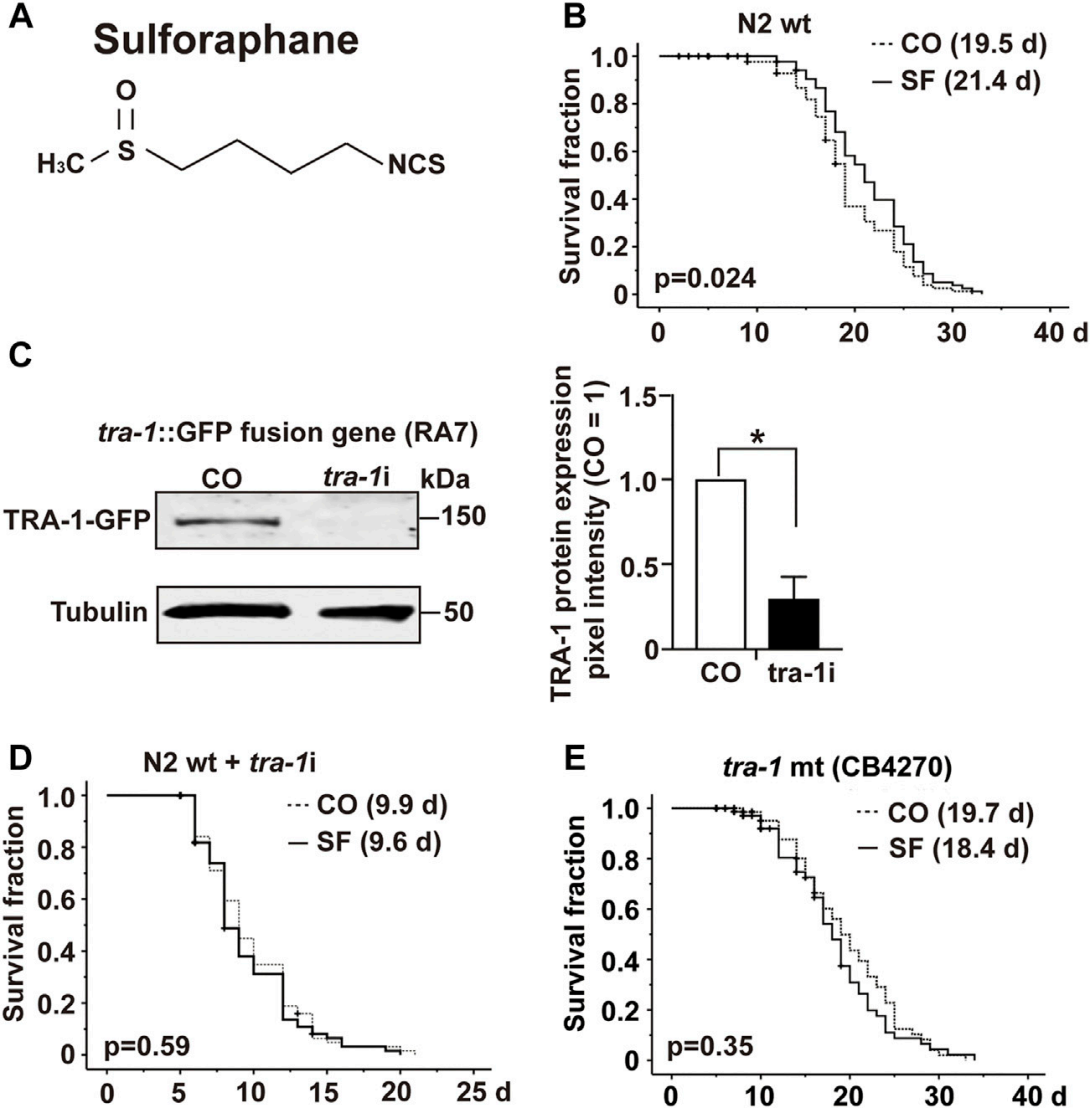
TRA-1 is involved in sulforaphane-induced longevity. **(A)** Schematic representation of the chemical structure of isothiocyanate sulforaphane. **(B)** Synchronized N2 wild-type *C. elegans* L4 larvae (*n* = 100/group) were transferred to NGM plates and maintained at 20°C. In addition to the regular *E. coli* OP50 food bacteria, the culture plates contained 100 µM sulforaphane (SF) or not (CO). The lifespan assay started from the late L4 larval stage, which was considered Day 0 (0 days). From Day 0 to Day 10, the *C. elegans* worms were transferred to new NGM plates daily. From Day 10, the worms were transferred every second day. The endpoint was defined as the day when all the worms were dead. In the Kaplan–Meier diagram, the numbers of living and censored worms are shown, and the log-rank test was used to evaluate the statistical significance. **(C)** Synchronized RA7 *C. elegans* L4 larvae harboring a *tra-1*-GFP fusion gene (*n* = 200/group) were fed *tra-1* RNAi-transduced *E. coli* HT115 bacteria (*tra-1*i*)* or nontransduced *E. coli* HT115 bacteria (CO). After 48 h, proteins were extracted, followed by Western blot analysis. A GFP-specific antibody was used to target the GFP part of the TRA-1 fusion protein. Tubulin served as a control for equal loading conditions. The protein sizes in kilodaltons (kDa) are provided (upper part). The relative pixel intensity was analyzed using ImageJ. The intensity of TRA-1-GFP expression was normalized relative to tubulin, and the amount obtained in control *C. elegans* worms was set to 1, as shown in the diagram on the right with the means, standard deviations and significance. **(D)** N2 wild-type *C. elegans* worms were treated as described above and analyzed by Kaplan–Meier survival analysis. The total number of worms in each experiment was 100. **(E)** Kaplan-Meier analysis was performed using the CB4270 *C. elegans* strain with a loss-of-function *tra-1* gene. The worms were treated as described above, but maintained at 15°C, as recommended by the CGC (compare Table 3). Individual *p* values are given, and *p* ≤ 0.05 was considered statistically significant.

To investigate the impact of sulforaphane, which is chemically a sulfur-containing isothiocyanate (Figure 1A), we fed synchronized L4 wild-type *C. elegans* larvae with *E. coli* OP50 food bacteria in the presence or absence of 100 µM sulforaphane. We chose this sulforaphane concentration because it is considered optimum to treat *C. elegans* (Qi et al., 2021). The number of worms was recorded daily until no more live worms could be found from Day 33 onwards. Afterward, a Kaplan–Meier survival analysis was performed. Sulforaphane significantly increased the mean lifespan of wild-type worms to 21.4 days compared with 19.5 days of untreated control worms, indicating a 9.9% longer survival (Figure 1B; Table 3).

**TABLE 3:** Sulforaphane effects on the lifespan of *C. elegans* strains.

*C. elegans* strains	Temperature (°C)	Fig. No.	Exp. No.	Sulforaphane (μM)	Mean lifespan ±SEM (days)	%, ^a^Mean increase (SF treatment)	*p* Value
N2	20	1B	1	0	16.19 ± 0.52	15.5	0.001
				100	18.70 ± 0.53		
			2	0	20.99 ± 0.52	8.9	0.037
				100	22.85 ± 0.47		
			3	0	19.47 ± 0.53	9.9	0.024
				100	21.40 ± 0.52		
N2 (*tra-1*i)	20	1D	1	0	9.89 ± 0.41	—	0.485
				100	9.55 ± 0.37		
			2	0	9.62 ± 0.31	—	0.839
				100	9.65 ± 0.31		
			3	0	9.09 ± 0.41	—	0.103
				100	10.24 ± 0.59		
CB4270	15	1E	1	0	19.70 ± 0.79	—	0.352
				100	18.43 ± 0.80		
			2	0	28.45 ± 0.89	—	0.710
				100	27.22 ± 0.90		
			3	0	27.88 ± 1.08	—	0.766
				100	28.20 ± 1.04		

a Mean lifespan: Corresponds to the mean lifespan as measured in the present study.

Summary and statistical analysis of sulforaphane effects on lifespan experiments. N2: Wild-type *C. elegans* strain; N2 (*tra-1*i): loss-of-function *tra-1 C. elegans* strain; CB4270: *tra-1*(−) mutant *C. elegans* strain. The mean lifespan values were analyzed by log-rank (Kaplan–Meier) statistical tests. *p* values < 0.05 were considered statistically significant. *p* values were calculated for individual experiments (*n* = 100). All statistical evaluations were calculated using SPSS 25.0. (−) not calculated because the standard deviation was >0.05.

SEM: standard error of the mean.

To examine the involvement of *tra-1* signaling, we inhibited TRA-1 by feeding *E. coli* HT115 bacteria transfected with *tra-1* RNAi constructs to the *C. elegans* RA7 strain, which expresses a TRA-1-GFP fusion protein (Mathies et al., 2004). After 48 h, the proteins were extracted (200 worms/group), and Western blot analysis was performed to ensure the effectiveness of RNAi-mediated inhibition of TRA-1. A representative Western blot image is shown along with a diagram of the pixel intensities of the TRA-1 band relative to the tubulin pixel intensity, which served as a control for equal conditions. This confirmed a significant inhibition of TRA-1-GFP expression of approximately 70% (Figure 1C).

Next, we set up a series of lifespan assays to examine the longevity of *tra-1(−)* mutant worms relative to the presence of sulforaphane. Kaplan–Meier survival analysis revealed that the mean lifespan of *tra-1* RNAi-treated nematodes that received sulforaphane was 9.6 days compared with 9.9 days of control worms, with no significant difference (Figure 1D). To further confirm the involvement of *tra-1* signaling in lifespan, the *tra-1(−)* mutant CB4270 worm strain was evaluated by Kaplan–Meier survival analysis, resulting in a survival of 18.7 days in the presence of sulforaphane and 19.7 days in untreated control worms, with no significant difference (Figure 1E). These data suggest that sulforaphane-induced prolongation of the lifespan in *C. elegans* is dependent on intact *tra-1* signaling.

### TRA-1 Expression is Upregulated by Sulforaphane

**FIGURE 2:**
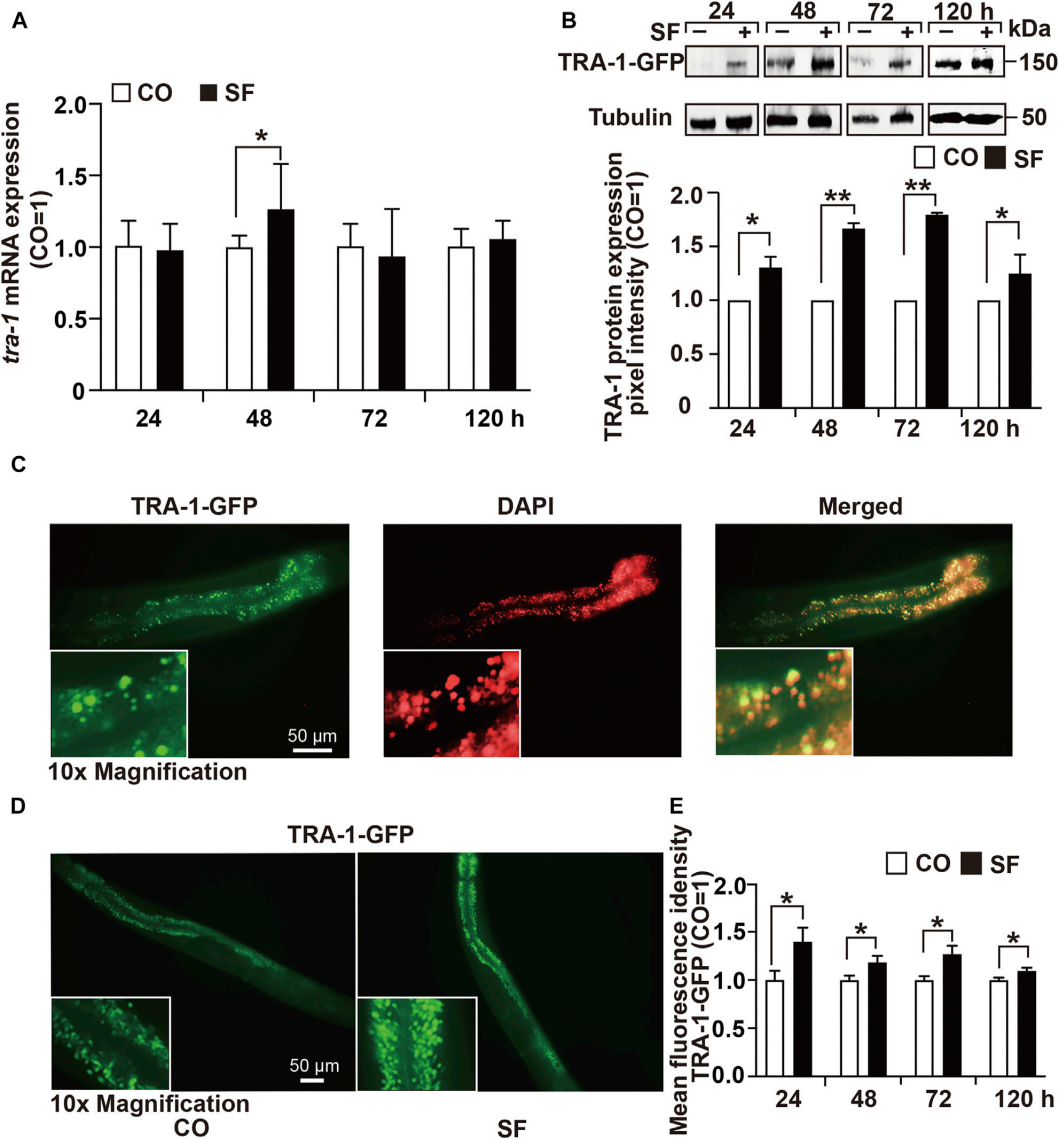
TRA-1 expression is upregulated by sulforaphane. **(A)** Synchronized N2 wild-type *C. elegans* L4 larvae (*n* = 200/group) obtained 100 µM sulforaphane (SF) in their growth plates or were left untreated in the control (CO). Next, 24, 48, 72, and 120 h later, the total RNA was extracted, and the expression of *tra-1* was detected by RT–qPCR. The expression of *tra-1* is given as the fold change and is normalized to the control group, whose expression was set to 1. **(B)** Synchronized RA7 *C. elegans* worms expressing a *tra-1*-GFP fusion gene were treated with sulforaphane (SF) or were left untreated in the control (CO). Proteins were extracted from 200 worms per group at 24, 48, 72, or 120 h after sulforaphane feeding, and the expression of TRA-1 was detected by Western blot analysis using a GFP-specific antibody. Detection of tubulin expression served as a control for equal loading conditions. The protein sizes in kilodaltons (kDa) are provided (upper part). ImageJ was used to evaluate the pixel intensity of each band, and the expression was normalized to Tubulin. Next, the values in the sulforaphane group were related to those in the control group, which were set to 1. **(C)** The RA7 *C. elegans* strain was used to examine the expression of TRA-1-GFP by detecting green GFP fluorescence by fluorescence microscopy. The cell nuclei were counterstained with DAPI. The images were taken at ×400 magnification. Insert: ×10 magnification. The scale bar indicates 50 µm. **(D)** RA7 *C. elegans* worms (*n* = 10/group) were fed 100 μM sulforaphane (SF) or not (CO), and 24, 48, 72, or 120 h later, the green spots, reflecting nuclear translocation of the TRA-1-GFP fusion protein, were detected by fluorescence microscopy. Images were taken at ×200 magnification. The inserts show a ×10 magnification. The scale bar indicates 50 µm. **(E)** The mean fluorescence intensities were quantified using ImageJ, and the statistical analysis is represented in the diagram. The data are shown as means ± SEM, as evaluated by Student’s t-test using SSPS 25. *p < 0.05, **p < 0.01.

To determine the effect of sulforaphane on *tra-1* expression, we fed synchronized wild-type worms in the presence or absence of sulforaphane. After 24, 48, 72, and 120 h, RNA was isolated, and *tra-1* expression was examined by RT–qPCR analysis. The level of *tra-1* was significantly upregulated at 48 h after sulforaphane feeding, whereas no obvious difference was observed at 24, 72, or 120 h (Figure 2A). Likewise, the *C. elegans* RA7 strain, which expresses a TRA-1-GFP fusion protein, was used to identify the effect of sulforaphane on TRA-1 protein expression. Synchronized RA7 *C. elegans* worms were fed with or without sulforaphane. Next, 24, 48, 72, and 120 h later, the proteins were extracted, and Western blot analysis was performed using a GFP-specific antibody. We observed that sulforaphane significantly induced TRA-1-GFP expression at each time point, as shown by a representative Western blot image and the evaluation of the mean pixel intensities by ImageJ and relation to the pixel intensities of the tubulin control (Figure 2B, Supplementary Figure S1).

Next, we examined native GFP fluorescence in untreated RA7 nematodes by fluorescence microscopy and counterstained the cell nuclei with DAPI. We observed spots with enhanced fluorescence intensity in intestinal nuclei, indicating the expression and nuclear accumulation of the TRA-1 transcription factor (Figure 2C). To further validate the sulforaphane-increased accumulation of the TRA-1-GFP fusion protein, we fed RA7 worms with or without sulforaphane. Thereafter, 24, 48, 72, and 120 h later, we counted the green fluorescent spots, indicating nuclear localization of the TRA-1-GFP fusion protein using fluorescence microscopy. The mean fluorescence intensities were assessed using ImageJ and revealed that sulforaphane further increased the nuclear accumulation of the TRA-1-GFP fusion protein at each time point (Figures 2D,E). These findings suggest that sulforaphane enhances TRA-1 expression.

### Sulforaphane Increases the Healthspan *via* TRA-1

**FIGURE 3:**
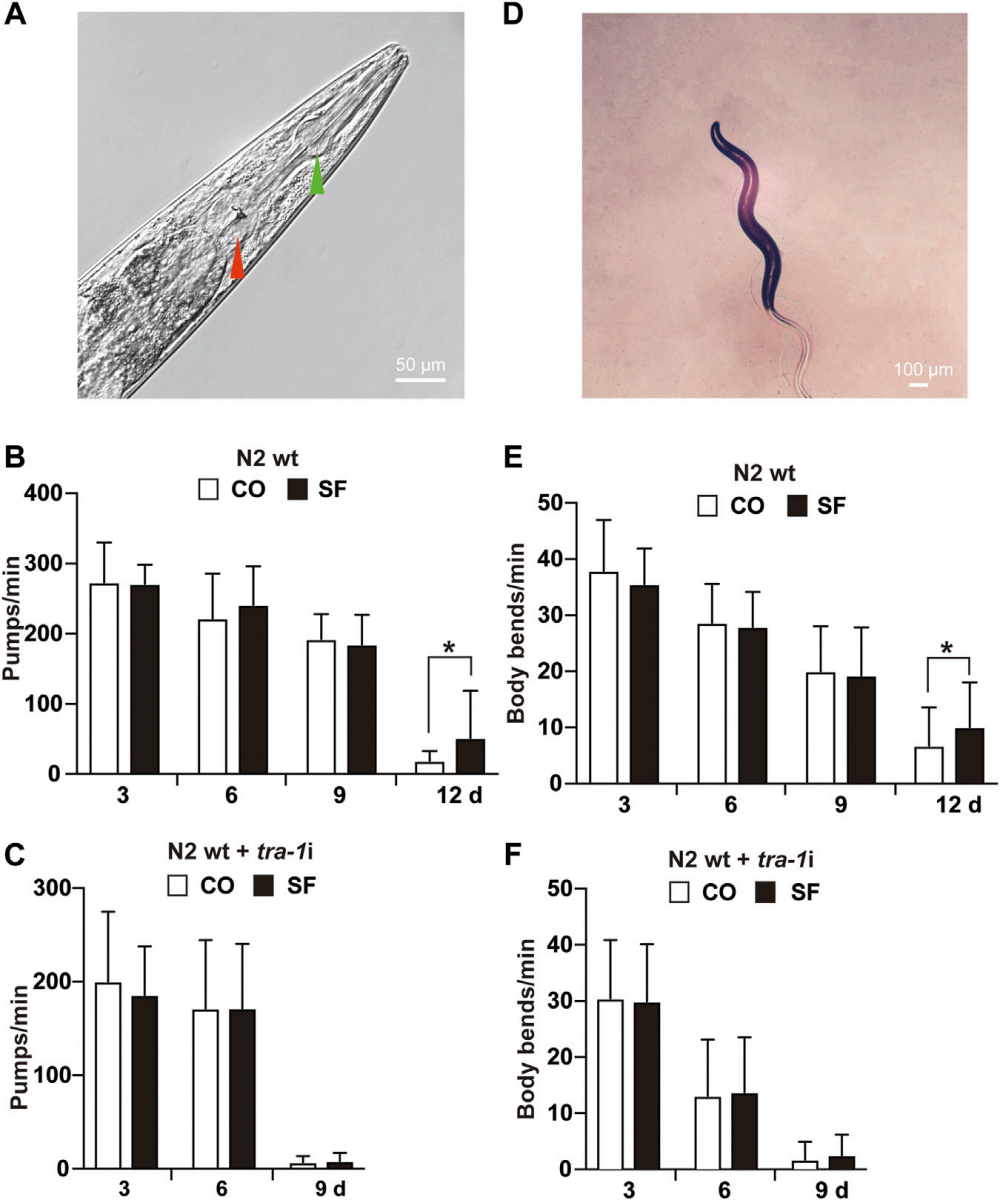
Sulforaphane increases the healthspan *via* TRA-1. **(A)** The *C. elegans* anterior pharynx (green arrow) and the terminal bulb (red arrow) are shown, which contract and relax synchronously during pharyngeal pumping. The scale bar indicates 50 µm. **(B)** Synchronized N2 wild-type *C. elegans* L4 larvae (*n* = 20/group) were exposed to 100 µM sulforaphane (SF) or not (CO) for 3, 6, 9, and 12 days. Afterward, pharyngeal pumping was detected under an inverted microscope. The pumping frequency per minute (Pumps/min) was calculated by evaluating the opening of the corpus, and the means, standard deviations and significance are shown in the diagram. **(C)** Synchronized N2 TJ356 *C. elegans* were fed *tra-1* RNAi-transfected *E. coli* HT115 (DE3) bacteria in the presence of 100 µM sulforaphane (SF) or not (CO). The pharyngeal pumping rate per minute (pumps/min) was detected at Days 3, 6, and 9. For each treatment, 20 worms were examined. **(D)** Image of a *C. elegans* nematode to visualize body bends. A change in the posterior bulb direction or regular swinging of the head was considered a body bend. The scale bar indicates 100 µm. **(E)** Synchronized N2 wild-type L4 larvae (*n* = 50/group) received a regular diet (CO) or were fed 100 µM sulforaphane on culture plates (SF). Next, 3, 6, 9, or 12 days later, the mobility of *C. elegans* was detected by counting the body bends per minute (body bends/min). (F) Synchronized N2 wild-type *C. elegans* were fed *tra-1* RNAi-transfected *E. coli* HT115 (DE3) bacteria in the presence of 100 µM sulforaphane (SF) or not (CO), and the body bends per minute were evaluated at the time points indicated. The data are expressed as means ± SEM. *p < 0.05, **p < 0.01.

To evaluate whether TRA-1 serves as a mediator of an increased healthspan after sulforaphane treatment, we analyzed the mobility and pharyngeal pumping rate of *C. elegans* because these features display age‐related declines (Collins et al., 2008). To assess appetite and food intake, the pumping frequency of the terminal pharyngeal bulb (Figure 3A) of each worm was documented by microscopy and video recordings at Days 3, 6, 9, and 12 after sulforaphane feeding. However, sulforaphane did not significantly alter the pharyngeal pumping frequency in younger worms at Days 3, 6, and 9; it significantly increased the frequency to 52 pumps/minute in older worms on Day 12 (Figure 3B). By contrast, untreated wild-type *C. elegans* worms exhibited 19 pumps/minute at Day 12, a time point at which the healthspan of worms was significantly decreased. Using *tra-1* RNAi-treated nematodes and examining the pharyngeal pumping rate in the presence or absence of sulforaphane, we observed no significant differences between the groups at any time point examined (Figure 3C). We would like to mention, that we tried to measure the pharyngeal pumping rate also at Day 12, which was, however, not possible, because *tra-1* RNAi transfection dramatically shortened the lifespan and at Day 12 almost all worms were dead, in both, the sulforaphane-treated and -untreated groups (compare Figure 1D). Interestingly, the healthspan of *tra-1* RNAi-treated *C. elegans* worms significantly decreased at Day 9, and the worms had a lower pumping rate from the beginning and exhibited an almost quiescent state with little pharyngeal pumping, reflecting the shorter healthspan.

Next, mobility was assessed by counting the number of body bends/minute (Figure 3D). Compared with untreated wild-type worms, a significant increase from 7 to 10 body bends/minute was observed in older worms at Day 12 after sulforaphane treatment, whereas no significant difference occurred in younger worms at Days 3, 6, and 9 (Figure 3E). By contrast, sulforaphane was ineffective in *C. elegans* with RNAi-mediated inhibition of TRA-1 expression (Figure 3F). These results indicate that TRA-1 expression is necessary for an enhanced healthspan following sulforaphane treatment.

### TRA-1 Mediates Sulforaphane-Induced DAF-16 Nuclear Translocation

**FIGURE 4:**
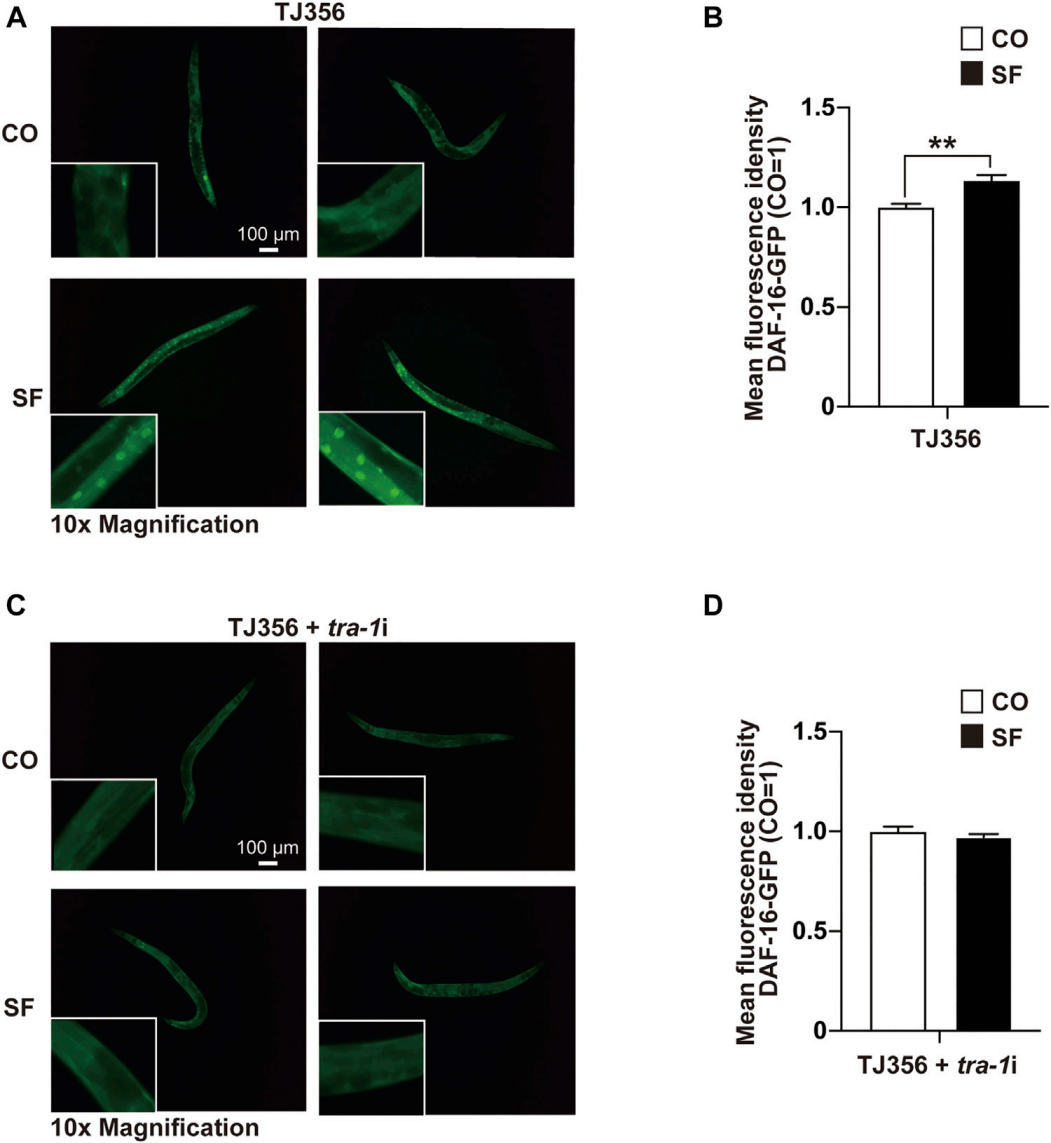
TRA-1 mediates sulforaphane-induced DAF-16 nuclear translocation. **(A)** Synchronized TJ356 *C. elegans* L4 larvae expressing a *daf-16*-GFP fusion gene were fed 100 µM sulforaphane (SF) or not (CO). Two days later, the nuclear translocation of DAF-16 was evaluated by detecting green fluorescent spots by fluorescence microscopy. Images were taken at ×100 magnification, and the inserts had a ×10 magnification. The scale bar indicates 100 µm. **(B)** The fluorescence intensity was quantified by ImageJ, and the means, standard deviations and statistical significance are shown in the diagram. **(C,D)** Synchronized TJ356 L4 larval nematodes were treated and evaluated as described above. The data are shown as means ± SEM. *p < 0.05, **p < 0.01.

To further elucidate the sulforaphane-induced signaling cascade, which mediates longevity and an increased healthspan, we focused on the transcription factor DAF-16 because its binding by TRA-1 was recently detected (Hotzi et al., 2018), and DAF-16 signaling was associated with the longevity of *C. elegans* (Lin et al., 2018). We used the *C. elegans* reporter strain TJ356, which expresses a DAF-16-GFP fusion protein (Lin et al., 2001). Synchronized TJ356 L4 larvae were fed with or without sulforaphane. After 48 h, the expression and cellular localization of the DAF-16-GFP fusion protein were examined by fluorescence microscopy. Sulforaphane-fed worms exhibited strong green fluorescent spots, indicating enhanced DAF-16 nuclear translocation, whereas the signal in control worms was lower (Figure 4A). This observation was confirmed by quantification of the fluorescence intensity using ImageJ, and the mean fluorescence intensities with standard deviations and significance are shown (Figure 4B). We confirmed these results by RNAi-mediated inhibition of TRA-1 expression in TJ356 *C. elegans* (Figures 4C,D). These data suggest that TRA-1 mediates sulforaphane-induced nuclear translocation of DAF-16.

### DAF-16 Is Required for TRA-1-Induced Lifespan and Healthspan

**FIGURE 5:**
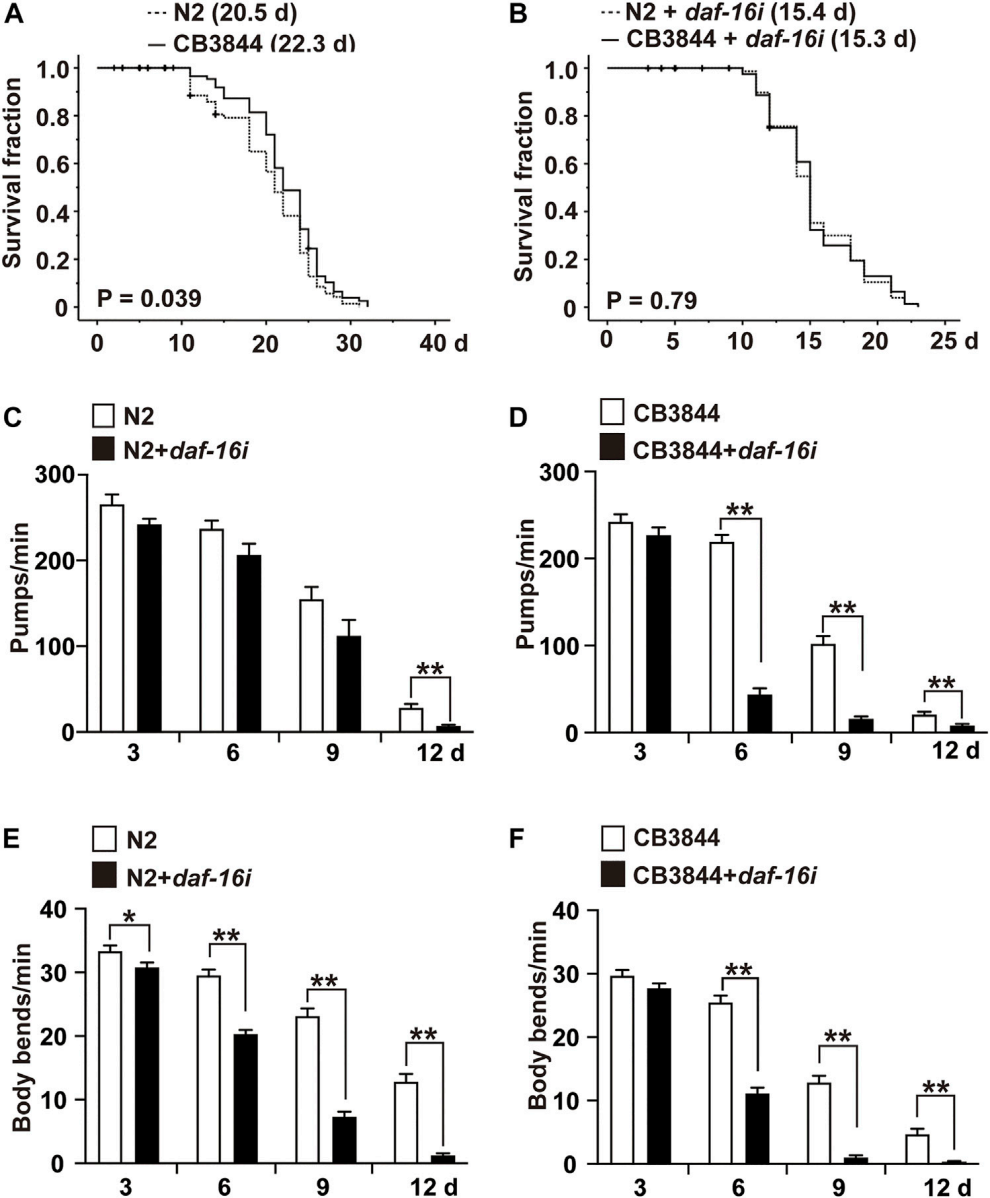
DAF-16 is required for TRA-1-induced lifespan and healthspan. **(A)** Synchronized N2 wild-type (N2) and CB3844 *C. elegans* L4 larvae (*n* = 100/group) were fed *E. coli* OP50 bacteria, followed by Kaplan-Meier analysis, as described above. **(B)** Synchronized N2 wild-type and CB3844 *C. elegans* L4 larvae (*n* = 100/group) were fed *E. coli* HT115 bacteria transfected with *daf-16* RNAi (*daf-16i*), and the mean lifespan was evaluated by Kaplan-Meier analysis. **(C)** Synchronized N2 wild-type *C. elegans* worms (*n* = 20/group) were fed *daf-16* RNAi-transduced *E. coli* HT115 bacteria (N2+*daf-16i*) or nontransduced *E. coli* HT115 bacteria (N2). After 3, 6, 9, and 12 days of treatment, the pharyngeal pumping rate of individual worms was detected for 60 s. **(D)** Synchronized CB3844 *C. elegans* were fed *E. coli* HT115 bacteria transfected with *daf-16* RNAi (CB3844+*daf-16i*) or untransfected *E. coli* HT115 bacteria (CB3844), and the pharyngeal pumping rate per minute (Pumps/min) was detected at Days 3, 6, 9, and 12. Twenty worms were examined in each group. **(E)** Synchronized N2 wild-type *C. elegans* (*n* = 50/group) were treated as described above, and the body bends/min were counted for 60 s at Days 3, 6, 9, and 12. **(F)** Synchronized CB3844 *C. elegans* were fed as described above, and the body bends/min were analysed.

To examine the relationship between TRA-1 and DAF-16 in longevity and healthspan, we evaluated the survival of CB3844 *fem-3* mutant worms that harbor a hyperactive TRA-1 protein (Hotzi et al., 2018). Compared with wild-type worms with an average lifespan of 20.5 days, the survival of CB3844 mutant worms was 22.3 days, corresponding to a longer survival of 8.9% (Figure 5A; Table 4). To determine whether DAF-16 is involved in TRA-1-mediated life prolongation, we inhibited *daf-16* by RNAi in wild-type and CB3844 mutant worms and performed Kaplan-Meier analysis. With inhibited *daf-16* expression, the CB3844 worms did not survive longer than the wild-type worms (Figure 5B), indicating that DAF-16 is required for TRA-1-induced longevity.

**TABLE 4:** Lifespan of *C. elegans* strains.

Temperature (°C)	Fig. No.	Exp. No.	C. elegans strains	Mean lifespan ±SEM (days)	%, *mean increase	*p* Value
20	5A	1	N2	19.72 ± 0.40	3.6	0.037
		—	CB3844	20.42 ± 0.48		
		2	N2	20.48 ± 0.60	8.9	0.039
		—	CB3844	22.29 ± 0.50		
		3	N2	21.74 ± 0.46	7.0	0.031
		—	CB3844	23.27 ± 0.54		
20	5B	1	N2 (*daf16*i)	15.35 ± 0.36	—	0.793
		—	CB3844(*daf16*i)	15.34 ± 0.38		
		2	N2 (*daf16*i)	15.55 ± 0.37	—	0.710
		—	CB3844(*daf16*i)	15.40 ± 0.38		
		3	N2 (*daf16*i)	16.24 ± 0.38	—	0.828
		—	CB3844(*daf16*i)	16.19 ± 0.39		

Mean lifespan: Corresponds to the mean lifespan as measured in the present study. The mean lifespan values were calculated by the log-rank (Kaplan–Meier) statistical test. *p* values less than 0.05 were considered statistically significant, whereas statistical significance was not calculated (-) when *p* values were >0.05. All statistical evaluations were calculated using SPSS 25. Each experiment included 100 *C. elegans* nematodes. SEM: standard error of the mean.

**FIGURE 6:**
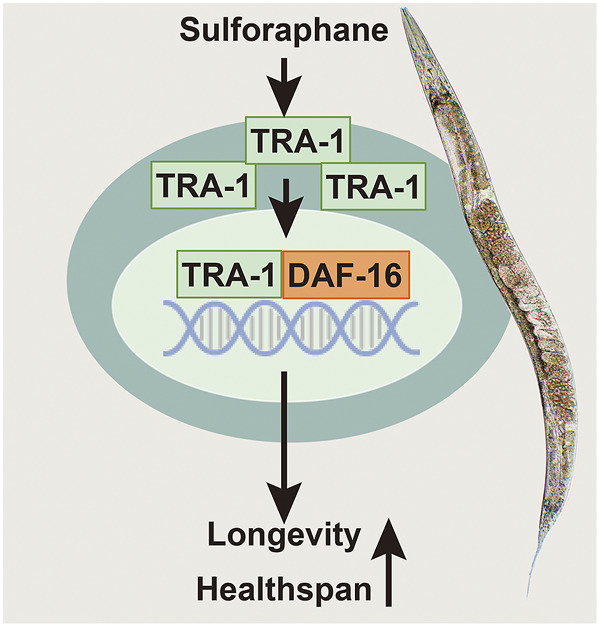
Scheme of sulforaphane-promoted lifespan and healthspan by inducing TRA-1 and DAF-16 nuclear translocation. Sulforaphane induces enhanced protein levels of the TRA-1 terminal transcription factor of the *C. elegans* sex-determination pathway. TRA-1 translocates to the nucleus, where it binds to DAF-16, which is the main target of the insulin/insulin-like growth factor signaling pathway, plays a fundamental role in lifespan, metabolism and stress responses and is related to the mobility and food intake of *C. elegans*. We assume that this scenario is responsible for the observed sulforaphane-mediated extended lifespan and improved healthspan of *C. elegans*.

Finally, we assessed whether DAF-16 may be required for the previously observed TRA-1-mediated prolonged healthspan, the expression of *daf-16* was inhibited by feeding *E. coli* HT115 bacteria transfected with *daf-16* RNAi, which resulted in a significant inhibition of *daf-16* RNA levels in N2 wild-type and CB3884 worms, as examined by RT–qPCR 48 h later (Supplementary Figure S1). The examination of pharyngeal pumping demonstrated that the pumping frequency significantly decreased from 28 to 7 pumps/minute at Day 12 in older wild-type worms, whereas no difference was observed in younger worms at Days 3, 6, and 9 (Figure 5C). This effect was even more pronounced in the *C. elegans* CB3844 strain following RNAi-mediated inhibition of *daf-16* because the pumping frequency already decreased not only at Day 12 but also at Days 6 and 9 (Figure 5D). Likewise, the inhibition of *daf-16* expression by RNAi in wild-type worms significantly reduced the body bends/minute at all time points examined (Figure 5E). The effects were even more pronounced following RNAi-mediated inhibition of *daf-16* in CB3844 worms (Figure 5F), as expected. These results imply that DAF-16 is a crucial downstream effector in TRA-1-mediated increased longevity and healthspan in *C. elegans* (Figure 6), although this conclusion requires further confirmation.

## Discussion

The present study hypothesized that the bioactive agent sulforaphane prolongs the lifespan and healthspan of *C. elegans* by inducing TRA-1 signaling, which mediates its effects by the downstream transcription factor DAF-16. Using *C. elegans* strains with hyperactive TRA-1 or loss-of-function mutations in *tra-1* and RNAi-mediated inhibition of *tra-1* or *daf-16* in wild-type *C. elegans*, we demonstrate that sulforaphane mediates its effects by inducing TRA-1 and downstream DAF-16 signaling. This finding was validated using Kaplan–Meier survival analysis, healthspan assays of pharyngeal pumping and the detection of body bending frequency.

According to our results, sulforaphane increased the average lifespan and significantly promoted the healthspan in the mid-life period of *C. elegans* at approximately 8–12 days of age, confirming our recent data, that demonstrated the regulation of DAF-16/FOXO signaling by sulforaphane and thereby an extension of the lifespan (Qi et al., 2021). However, it remained unclear by which upstream step sulforaphane leads to modification of DAF-16 signaling. Our new findings in the present study demonstrate that sulforaphane induced the expression of the *tra-1* gene that in turn regulates DAF-16 signaling. These findings are consistent with previous studies showing a benefit of sulforaphane in cancer chemoprevention and human health, such as anti-inflammatory and anti-atopic allergic responses (Brown et al., 2015; Bayat Mokhtari et al., 2018; Sidhaye et al., 2019). Conversely, we observed that the loss of *tra-1* expression or its function shortened the lifespan and decreased the healthspan, which could not be reversed by sulforaphane. Therefore, we assume that TRA-1 is a crucial modulator of sulforaphane-promoted longevity and healthspan in *C. elegans.* This assumption was confirmed by Western blot analysis, which demonstrated upregulated TRA-1 protein expression by sulforaphane. Additionally, we found that the average lifespan of *C. elegans* with hyperactive TRA-1, because of a *fem-3* mutation, increased by 8.9%. Our data agree with those of a previous study implying a longer survival of hermaphroditic *C. elegans* worms with a higher TRA-1 expression level than that of male worms (Johnson and Wood, 1982). By contrast, we did not detect sulforaphane-induced upregulation of *tra-1* RNA levels. This observation might be explained by the data of a recent report demonstrating that the regulation of *tra-1* occurs posttranscriptionally in male and hermaphroditic *C. elegans* nematodes (Zarkower and Hodgkin, 1992).

Furthermore, we observed that sulforaphane promoted the nuclear translocation of TRA-1 and its downstream target DAF-16. Naturally, the sulforaphane-induced translocation of DAF-16 was prevented in *tra-1* (−) mutant *C. elegans* nematodes and following RNAi-mediated *tra-1* inhibition in TJ356 worms. Additionally, we observed that food intake and mobility significantly decreased in *C. elegans* worms with dysfunctional *daf-16*. This finding is not surprising because DAF-16 is the main target of the insulin/insulin-like growth factor signaling pathway (Murphy and Hu, 2013), plays a fundamental role in lifespan, metabolism and stress responses and is related to the mobility and food intake of *C. elegans* (Thomas, 1999; Ryu et al., 2016; Lin et al., 2018; Wang et al., 2019; Aghayeva et al., 2021). These facts may explain our observation of decreased pharyngeal pumping and body bending rates in TRA-1 hyperactive *C. elegans* mutant worms after RNAi-mediated *daf-16* inhibition. The fact that sulforaphane fails to promote longevity in absence of *tra-1* but nevertheless does not significantly upregulate *tra-1* mRNA expression is indeed intriguing. Therefore, it may be plausible that mRNA expression of *tra-1* is required for sulforaphane to exert its effect. However, we cannot exclude that *tra-1* may very well upregulate another unidentified mediator beside *daf-16*.

Despite our promising findings, there are still limitations of our study. For example, we only performed Kaplan-Meier survival analysis and healthspan assays to investigate the interaction of TRA-1 and DAF-16, and we did not perform e.g. pull-down assays to demonstrate a direct TRA-1-DAF-16 interaction. However, a recent study described two conserved TRA-1 binding sites in the *daf-16* promoter (Hotzi et al., 2018), suggesting that the binding of TRA-1 to DAF-16 is necessary for the sulforaphane-induced nuclear translocation of DAF-16. These findings prompted us to speculate that DAF-16 is a downstream target in TRA-1-induced heathy aging in *C. elegans*. However, this suggestion still requires further confirmation.

Finally, we even suspect that our data in *C. elegans* may be of relevance to human health, because many epidemiological and pharmacological studies have shown that sulforaphane consumption is associated with a reduced risk of disease in humans. Recent data have demonstrated the effects of sulforaphane on metabolism-related disorders and concluded that sulforaphane improves glycemic control in type 2 diabetes and ameliorates the changes associated with diabetic nephropathy (Wu et al., 2015; Axelsson et al., 2017). Furthermore, the regulation of lifespan by the insulin/insulin-like growth factor signaling pathway is highly conserved in nematodes and humans (Tazearslan et al., 2011). Interestingly, variants of FOXO3A—the mammalian DAF-16 homolog—are more frequent in centenarians than in 90-year-olds, as reported in a German cohort study (Flachsbart et al., 2009). These previous studies in humans and our results obtained in the *C. elegans* model prompt us to speculate that sulforaphane might extend the lifespan also in humans. However, an open question is how much sulforaphane—e.g., in the form of broccoli or its sprouts—should be ingested by humans to cause antiaging or anticancer effects. Some reports, including a prospective study, have observed a reduced risk of aggressive prostate cancer by a high intake of more than one serving of sulforaphane-rich broccoli or cauliflower per week (Kirsh et al., 2007). A recent pilot patient study in advanced pancreatic cancer used a daily intake of 90 mg of sulforaphane in the form of broccoli sprouts and observed positive, although nonsignificant, effects on the survival of cancer patients (Lozanovski et al., 2019).

## Conclusion

Our study demonstrates that sulforaphane initiates TRA-1 signaling via its downstream target DAF-16 to prolong the lifespan and healthspan of *C. elegans*. These data provide a promising hint regarding the suitability of sulforaphane as a novel anti-aging drug and *tra-1* as a novel target in anti-aging and disease prevention strategies.

## Data Availability

The original contributions presented in the study are included in the article/Supplementary Material, further inquiries can be directed to the corresponding author.
