# Supplementary material for: Sulforaphane Targets TRA-1/GLI Upstream of DAF-16/FOXO to Promote C. elegans Longevity and Healthspan
Source: Front Cell Dev Biol. 2021 Dec 3;9:784999. doi: 10.3389/fcell.2021.784999 (PMC8678450; doi:10.3389/fcell.2021.784999)
Supplement: Supplementary file 1 [file DataSheet1.pdf]

Supplemental Material

Sulforaphane targets TRA-1/GLI upstream of DAF-16/FOXO to promote *C. elegans* longevity and healthspan

Huihui Ji, Zhimin Qi, Daniel Schrapel, Monika Le, Yiqiao Luo, Bin Yan,
Jury Gladkich, Michael Schäfer, Li Liu, Ingrid Herr

Table of Content

Supplemental Figures with Legends

Figure S1 Original crude Western blot images 2

Figure S2 Downregulation of *daf-16* by feeding *E. coli* bacteria transfected with *daf-16 RNAi* constructs...3

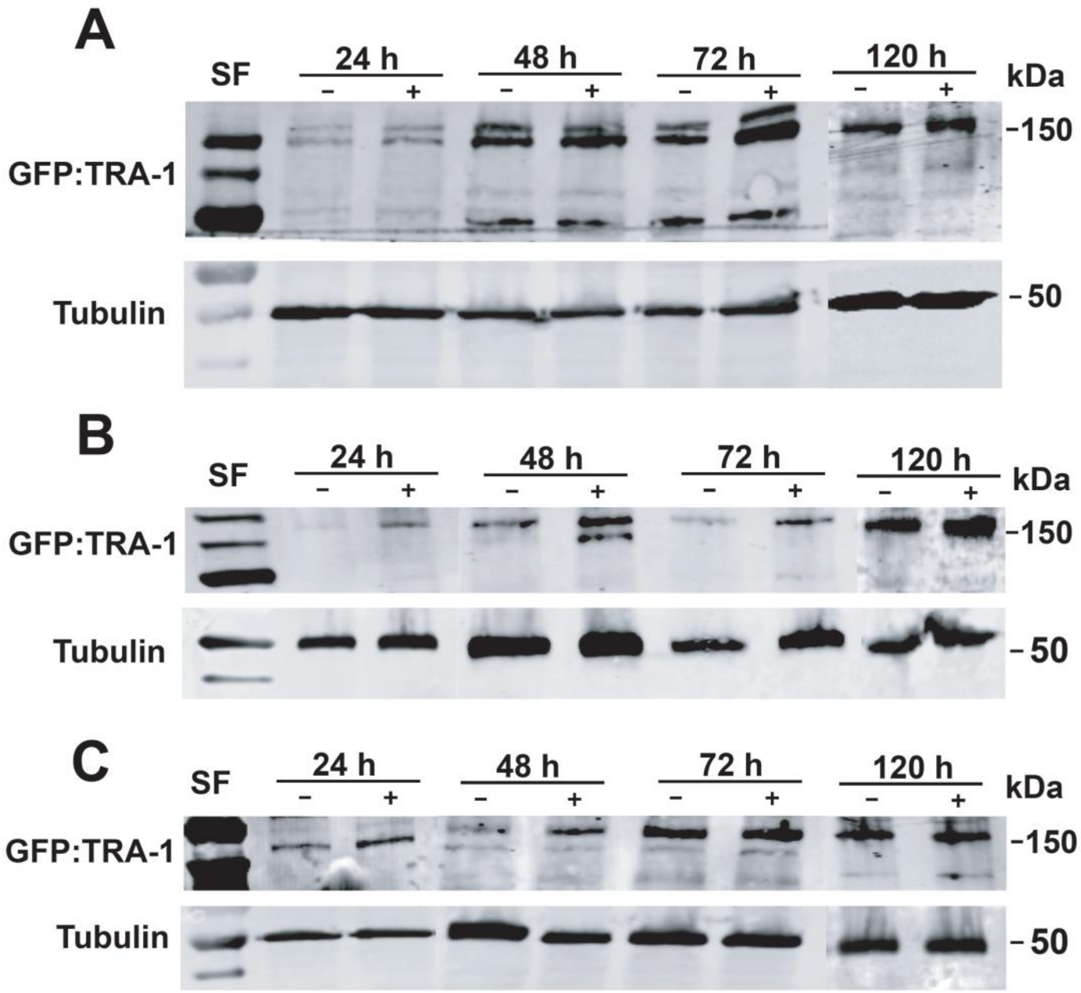

Figure S1. Original crude Western blot images. These images belong to Fig. 2B and molecular weight markers are shown.

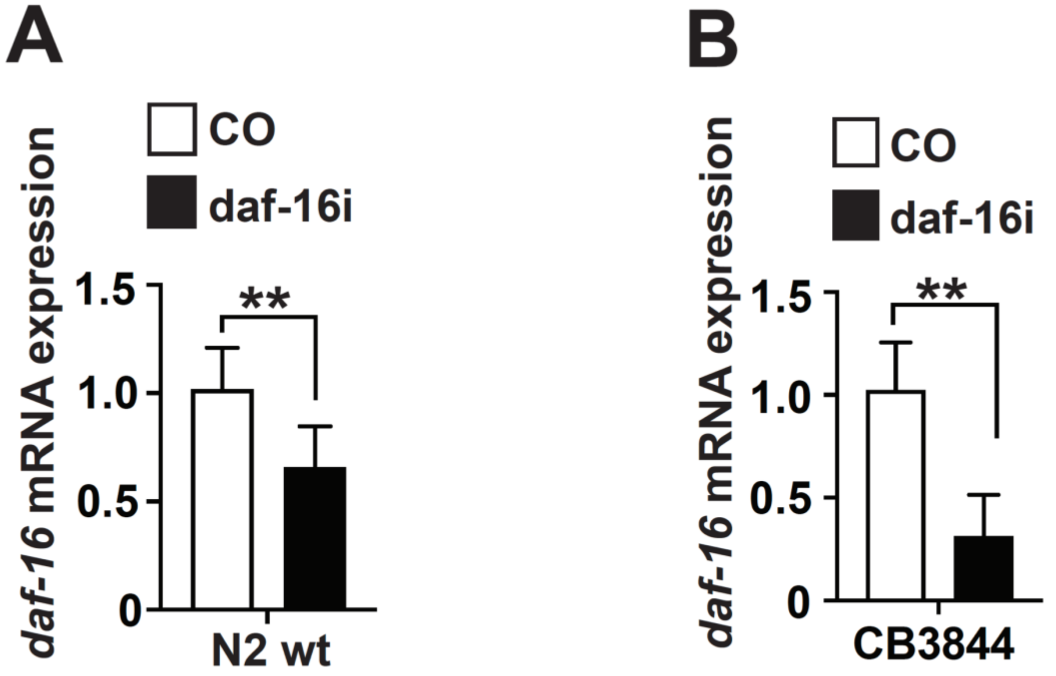

Figure S2. Downregulation of daf-16 expression by feeding *E. coli* bacteria transfected with daf-16 RNAi constructs. (A, B) Synchronized N2 wild-type and CB3844 *C. elegans* L4 larvae (n=100/group) were fed with *E. coli* HT115 bacteria transfected with *daf-16* RNAi (*daf-16i*), or with non-transfected *E. coli* HT115 bacteria (CO). After 48 hours, the expression of daf-16 was examined by RT-qPCR. The expression of *daf-16* is given as fold change and is normalized to the control group, whose expression was set to 1.
